# Glaucoma as a Tauopathy—Is It the Missing Piece in the Glaucoma Puzzle?

**DOI:** 10.3390/jcm12216900

**Published:** 2023-11-02

**Authors:** Maria Laura Passaro, Francesco Matarazzo, Gianmarco Abbadessa, Antonio Pezone, Antonio Porcellini, Fausto Tranfa, Michele Rinaldi, Ciro Costagliola

**Affiliations:** 1 Department of Neurosciences, Reproductive Sciences and Dentistry, University of Naples “Federico II”, 80131 Naples, Italy; marialaura.passaro@unina.it (M.L.P.); fausto.tranfa@unina.it (F.T.); ciro.costagliola@unina.it (C.C.); 2 Pineta Grande Hospital, 81030 Castel Volturno, Italy; francesco.matarazzo@unina.it; 3 Division of Neurology, Department of Advanced Medical and Surgical Sciences, University of Campania Luigi Vanvitelli, 80138 Naples, Italy; gianmarcoabbadessa@gmail.com; 4 Department of Biology, University of Naples “Federico II”, 80126 Naples, Italy; antonio.pezone@unina.it (A.P.); antonio.porcellini@unina.it (A.P.)

**Keywords:** glaucoma, neurodegeneration, tauopathy, oxidative damage

## Abstract

Glaucoma is a chronic neurodegenerative disorder affecting the visual system which can result in vision loss and blindness. The pathogenetic mechanisms underlying glaucomatous optic neuropathy are ultimately enigmatic, prompting ongoing investigations into its potential shared pathogenesis with other neurodegenerative neurological disorders. Tauopathies represent a subclass of neurodegenerative diseases characterized by the abnormal deposition of tau protein within the brain and consequent microtubule destabilization. The extended spectrum of tauopathies includes conditions such as frontotemporal dementias, progressive supranuclear palsy, chronic traumatic encephalopathy, and Alzheimer’s disease. Notably, recent decades have witnessed emerging documentation of tau inclusion among glaucoma patients, providing substantiation that this ocular disease may similarly manifest features of tauopathies. These studies found that: (i) aggregated tau inclusions are present in the somatodendritic compartment of RGCs in glaucoma patients; (ii) the etiology of the disease may affect tau splicing, phosphorylation, oligomerization, and subcellular localization; and (iii) short interfering RNA against tau, administered intraocularly, significantly decreased retinal tau accumulation and enhanced RGC somas and axon survival, demonstrating a crucial role for tau modifications in ocular hypertension-induced neuronal injury. Here, we examine the most recent evidence surrounding the interplay between tau protein dysregulation and glaucomatous neurodegeneration. We explore the novel perspective of glaucoma as a tau-associated disorder and open avenues for cross-disciplinary collaboration and new treatment strategies.

## 1. Introduction

The term ‘glaucoma’ encompasses a group of ocular conditions characterized by progressive optic nerve damage associated with a gradual visual field loss due to a slowly progressive degeneration of retinal ganglion cells (RGCs) [1]. Although intraocular pressure (IOP) reduction represents the only proven treatable risk factor, in some glaucomatous patients, RGC loss may continue despite the reduction in IOP. Thus, independently or in addition to IOP, other factors can individually or collectively contribute to the death of retinal ganglion cells and optic nerve fibers in glaucoma [1].

In recent years, significant efforts have been made to fully understand the mechanisms underlying the pathophysiology of glaucoma. Even if it is unquestionable that much progress has been made, and that glaucoma is now recognized as a full-fledged neurodegenerative disease, several mechanisms underlying glaucomatous damage remain unknown.

Recently, the hypothesis of a correlation between glaucoma and protein tau (commonly associated with neurodegenerative disorders) has gained significant attention, suggesting that tau may also play a role in glaucomatous neurodegeneration [2,3,4,5].

Tau is a microtubule-stabilizing protein, and its function is intricately regulated through phosphorylation at numerous sites, thereby influencing its function and turnover based on its phosphorylation status. It is mainly expressed in neurons, playing a crucial role in the assembly of tubulin monomers into microtubules and constituting the neuronal microtubule network [6].

Hyperphosphorylation of tau depresses its biological activity, causing both microtubule instability and the formation of aberrant fibrillar polymers. Moreover, changes in microtubule organization can result in a disorganization of mitochondria or lysosomes [7,8,9,10].

A dysregulation in the phosphorylation of tau protein is responsible for a group of neurodegenerative disorders referred to as tauopathies [11].

This review endeavors to investigate the potential role of the tau protein in the pathogenesis of glaucoma and its effects on retinal ganglion cells (RGCs), and the mechanisms of the optic nerve response to glaucomatous damage.

A deep understanding of these processes may result in the development of novel targeted therapeutic strategies to halt or retard glaucoma progression and to improve the visual outcome of patients. Among the potential avenues for exploring new treatments, there are strategies targeting various disease mechanisms, such as MAPT expression suppression, alternative splicing and post-translational modifications regulation, microtubule stabilization, aggregation inhibition, passive or active immunization, and innovative approaches related to genome integrity preservation.

In this review, we conducted an extensive literature search using the PubMed, Scopus, and Cochrane databases. The search strategy involved a combination of keywords to capture relevant studies, including “glaucoma AND tau,” “glaucoma AND neurodegeneration,” “glaucoma AND tauopathies,” “tau AND retinal ganglion cells,” and “tau AND optic nerve.” The identified articles were reviewed and analyzed to provide a comprehensive overview of the current state of knowledge in this field.

## 2. Ocular Structures and Glaucoma Pathophysiology

Although the pathophysiology of glaucoma is not entirely known, the loss of RGCs is known to be correlated with IOP. The IOP is balanced by the ciliary body’s production of aqueous humor (AH) and its drainage. Historically, aqueous humor outflow pathways have been classified into two main categories, the conventional (trabecular meshwork (TM)) and unconventional (uveoscleral) pathways [12]. In the conventional route, AH departs from the anterior chamber through the TM. Subsequently, it passes through the juxtacanalicular tissue (JCT), a loose connective tissue with an irregular network in which TM cells are surrounded by fibrillar elements of the extracellular matrix (ECM). Then, it is conveyed into the Schlemm canal, an endothelium-lined vessel that encircles the cornea, presenting features similar to both blood and lymphatic vasculature [13,14]. Then, through the network of collector channels continuous with the venous system, AH leaves the eye. To a lesser extent compared to this route discussed above, a portion of aqueous humor is drained through the unconventional pathway. In this pathway, AH is drained through the interstices of the ciliary muscle and ultimately through the choroid and sclera [14]. However, recent studies have shed light on the existence of previously unrecognized additional outflow routes. Notably, these newly identified pathways include the transcleral outflow pathway and uveolymphatic pathway, suggesting a more intricate and multifaceted network of aqueous humor drainage than was previously understood [15]. While in open-angle glaucoma there is an increased resistance to AH outflow, the drainage is typically obstructed in angle-closure glaucoma. In patients with elevated IOP, mechanical stress and tension on the posterior eye structures, particularly the lamina cribrosa, can lead to damage to retinal ganglion cells and axonal transport disruption [16,17]. Such changes can occur early in the development of glaucoma, leading to the accumulation of vesicles, and microtubule and neurofilament disorganization, in the prelaminar and postlaminar regions [12]. Glaucomatous optic neuropathy may also occur when IOP levels are within the normal range. This form is known as normal tension glaucoma, and the probable pathogenesis is vascular [18]. Other factors which may contribute to glaucoma pathogenesis include impaired microcirculation, altered immunity, and excitotoxicity [12].

## 3. The Role of Tau in Neurodegenerative Disorders

‘Tauopathies’ is an umbrella term referring to a group of neurodegenerative diseases characterized by the deposition of tau protein within the brain in the form of neurofibrillary tangles and paired helical filaments [19]. The deposition occurs primarily in neurons, but also in glial cells and in the extracellular space [19].

Under physiological conditions, the tau protein is primarily localized in axons [20] and plays a crucial role in microtubule assembly, stability, and dynamics [21,22]. However, several other functions of tau protein have been proposed [23,24].

It is encoded by the microtubule-associated protein tau (MAPT) gene [20]. Through alternative splicing of the exons 2, 3, and 10, the pre-mRNA can generate six distinct isoforms. Exon 10 encodes for the second microtubule-binding repeat domain (MTBD) in the C-terminal region [25]. The inclusion or exclusion of exon 10 determines the classification of these isoforms, based on the presence of three or four repeats of MTBDs [25]. Consequently, tau isoforms are categorized as either 3R or 4R isoforms. Both 3R and 4R isoforms are equally represented under physiological conditions in the adult human brain [26].

Tau binds to microtubules via repeat MTBDs in the C-terminus, and its phosphorylation plays a critical role in regulating its affinity to microtubules [27]. Hyperphosphorylation of tau, along with other tau alterations such as methylation, overexpression, and post-translational modifications other than phosphorylation, can lead to a decrease in binding affinity [28]. This reduction in binding affinity ultimately results in tau deposition and destabilization of microtubules [28].

Despite extensive research, the exact mechanism by which tau contributes to neurodegeneration remains to be fully elucidated. Various mechanisms have been proposed, including gain of function, loss of function, and mislocalization of tau [29]. These different hypotheses suggest that tau may exert its pathological effects through diverse mechanisms, further highlighting the complexity of its involvement in neurodegenerative processes.

Furthermore, it is important to note that alterations in other protein pathways could also play a role in the pathogenesis of tauopathies. The brain changes associated with Alzheimer’s disease can arise from an intricate interplay involving the abnormal accumulation of tau and beta-amyloid proteins, alongside various other factors. More precisely, abnormal tau tends to accumulate in particular regions of the brain associated with memory function. On the other hand, beta-amyloid tends to aggregate into clumps or plaques that develop in the spaces between neurons [30].

Based on the predominant tau isoform found in the aggregates, tauopathies are pathologically classified into three main categories: 3R tauopathies (frontotemporal dementia (FTD)), 4R tauopathies (progressive supranuclear palsy (PSP), corticobasal degeneration (CBD), argyrophilic grain disease (AGD), and globular glial tauopathy (GGT)), and 3R/4R tauopathies (Alzheimer’s disease (AD), chronic traumatic encephalopathy (CTE)) and primary age-related tauopathy (PART) [19].

Clinical manifestations of tauopathies vary depending on the specific type and stage of the disease. Common symptoms observed across various tauopathies include memory impairment, behavioral changes, executive dysfunction, and motor impairment [19]. Motor symptoms, such as parkinsonism with rigidity and bradykinesia, are frequently seen in 4R tauopathies like PSP, corticobasal syndrome (CBS), AGD, and GGT [19,31].

The behavioral variant of frontotemporal dementia (bvFTD) is distinguished by isolated behavioral changes as its characteristic early manifestation, while the non-fuent variant primary progressive aphasia (nfvPPA) and semantic-variant primary progressive aphasia (svPPA) are typified by isolated speech disturbances [32,33,34]. Conversely, single or multidomain amnesia is identified as the predominant symptom marking the onset of Alzheimer’s disease (AD) [33]. However, as the disease progresses, these manifestations progressively overlap [19]. According to a cross-sectional study involving 310 patients diagnosed with frontotemporal lobar degeneration (FTLD), it was found that 62% of the participants fulfilled the diagnostic criteria for multiple syndromes [32]. In advanced stages, individuals with tauopathies may experience difficulties with daily activities, impaired mobility, and significant functional impairment.

Concerning the clinicopathological correlation, a previous study [35] has delineated a stepwise progression of tau pathology through four defined phases in FTD. Beginning with Phase I, tau deposits initially emerge in the frontotemporal limbic/neocortex and angular gyrus. By Phase IV, even the primary visual cortex begins to exhibit a mild tau accumulation. Clinically, these tau depositions manifest in distinct ways. The majority of patients, right from the early tau accumulation stages, displayed hallmark features of bvFTD, especially social comportment disorders. This suggests that the initial tau accumulations in limbic and paralimbic regions, crucial for social behavior and emotion regulation, could be directly associated with these behavioral manifestations. Intriguingly, despite significant early tau presence in the hippocampus, a memory center, episodic memory difficulties often emerged as a later clinical symptom. This brings forth the possibility that other factors, possibly the involvement of prefrontal regions, might modulate the relationship between tau pathology and memory impairment. Adding another layer of complexity, one study’s neuroimaging findings [35] pinpointed early degeneration in the anterior insula and cingulate cortex, areas critical for emotion processing, further emphasizing the early behavioral changes seen in bvFTD. As tau pathology extended, the range of clinical symptoms widened, mirroring the affected regions. However, it is essential to underscore that tau pathology’s relationship with clinical manifestations is not always linear. Factors like specific brain regions’ resilience, compensatory neural mechanisms, and other concurrent pathological processes can modulate this relationship. In essence, while tau pathology’s progression in PiD is clear, translating this to clinical symptoms presents a nuanced picture, revealing the intricate interplay of neurodegenerative mechanisms in PiD.

Looking at the 4R tauopathies, PSP’s [36] neuronal tau pathology is evident in subcortical areas, whereas astroglial tau emerges prominently in the neocortex and striatum. This specific pathology manifests as atrophy, especially in the subthalamic nucleus and brainstem, which greatly affects motor functions. CBD [37], while sharing similarities with PSP, primarily impacts the frontal regions of the brain with a strong asymmetry, causing distinct motor and cognitive issues. The disease pathology is evident in regions like the frontoparietal cortices, striatum, and the amygdaloid body. AGD [38] is recognized by its 4R tau composition, with the presence of argyrophilic grains that significantly relate to cognitive decline. GGT [39], on the other hand, offers a complex profile marked by a predominance of 4R tau, especially in the glial cells.

Finally, moving to 3R and 4R tauopathies, AD [40] is characterized by an equal distribution of 3R and 4R tau, with neurofibrillary tangles (NFTs) and amyloid plaques forming the hallmarks of the disease. These pathological features result in cognitive decline and are intricately tied to the interactions between tau and Aβ. CTE [41], on the other hand, presents a mixed expression of 3R and 4R tau that undergoes a transition as the disease progresses. The tau deposits, mainly found in regions like the neocortex and hippocampus, relate closely to the severity of the disease and its differentiation from AD. Lastly, PART [42], bearing AD-like NFTs and minimal Aβ plaques, triggers cognitive decline mainly localized to regions such as the temporal lobes and basal forebrain.

Advancements in the in vivo detection of tau have been made through neuroimaging, cerebrospinal fluid, and blood markers, offering biomarkers for early diagnosis, disease progression prediction, therapy monitoring, and clinical trial participant selection [12,43,44].

## 4. Tau Protein’s Role in Glaucoma: A Closer Look at the Evidence

Glaucoma is a neurodegenerative disease of the central nervous system characterized by the progressive degeneration of RGCs, damaging the optic nerve, and resulting in irreversible visual loss [12,16,45,46,47].

Despite high IOP being amply demonstrated to be the most significant known risk factor for glaucoma development [48,49], the mechanism by which elevated pressure damages RGCs is still a matter under investigation [5].

At present, the hypothesis that retinal and brain degeneration share pathogenic mechanisms is gaining more traction, and the role of tau protein in the pathogenesis of glaucoma is a subject of ongoing debate [50,51,52,53,54].

*Tau intraretinal accumulation*. Several studies have been conducted in animal models transgenic for P301S tau demonstrating the presence of phosphorylated tau protein in the retina. In 2011, Gasparini et al. [50] evaluated the retina of the mouse line transgenic for P301S tau, reporting a hyperphosphorylated transgenic tau accumulation in the nerve fiber layer (NFL). Further, from 2 months of age, the authors observed aggregation into filamentous inclusions in RGCs, with axonopathy and accumulation of hyperphosphorylated tau in the NFL preceding inclusion formation [50]. Then, Schon et al. monitored the development of retinal tau pathology on a single-cell level in vivo, demonstrating an increase in the number of RGCs containing fibrillar FSB-positive tau aggregates [55]. Moreover, they showed hyperphosphorylated tau in the retinas of different human tauopathies [56].

*Symptoms and eye structural changes linking neurodegenerative diseases and glaucoma.* Evidence from recent years shows how patients with AD often develop visual impairments, frequently associated with abnormalities in the eye [56]. Visual changes reported in the early stages of AD include difficulties in reading and finding objects, visual field loss, altered depth perception, abnormal color discrimination, and contrast sensitivity [57,58,59]. Although all of these issues were originally believed to originate exclusively from pathological cortex changes related to the underlying neurological disease, modern ocular imaging techniques have made it possible to prove a correlation between visual symptoms and structural ocular changes [60]. Ocular abnormalities found include a reduction in the number of optic nerve head axons, a reduced number of RGCs, and a decrease in the thickness of the retinal nerve fiber layer (RNFL) [61,62,63,64,65,66] and thinning of the RGC layer [67]. These findings overlap with the actual tool for glaucoma diagnosis, as the same parameters are currently used for the early analysis of glaucomatous damage [68]. In preperimetric glaucoma, which represents the early stage of the disease before significant visual field damage occurs, ganglion cell complex (GCC) parameters emerge as the first indicators of glaucomatous progression, demonstrating their exceptional utility in detecting preperimetric glaucomatous damage and effectively distinguishing preperimetric subjects from controls [69]. In cases of established glaucoma, RNFL thickness consistently exhibits reductions when compared to controls, while GCC parameters are also reduced in this established stage of glaucoma. Spectral-domain OCT parameters have displayed a similar capacity for diagnosing moderate and severe glaucoma, with their diagnostic accuracy increasing proportionally with the advancement of the disease [69,70,71,72].

An increased occurrence rate of glaucoma in patients with AD has been demonstrated in various studies [73,74]. In 2017, den Haan et al. conducted a meta-analysis comparing retinal thickness in AD, mild cognitive impairment, and healthy controls in 25 studies, finding that AD patients had a lower peripapillary RNFL and decreased total macular thickness compared to healthy controls [75].

*Glaucoma and cognitive impairment.* Recent advancements in our understanding of cognitive impairment in individuals with glaucoma have shed new light on the intricate relationship between ocular health and cognitive function. Patients affected by glaucoma showed lower cognitive scores and were associated with an increased risk of dementia [76,77,78]. Maurano et al. reported that glaucoma patients had a reduction in cognition similar to the values reported in the literature for patients with AD [79]. These findings are highly intriguing, once again providing valuable avenues for framing the neurodegenerative aspect of glaucoma. However, it is important to note that these studies are all based on aggregate patient data, and there is a lack of data explaining the differences between patients with glaucoma and cognitive disorders and those with glaucoma who perform within normal ranges on cognitive tests.

Lee et al. investigated the relationship between the lamina cribrosa thickness (LCT) and cognitive function in glaucoma patients, finding that a more pronounced impairment of cognitive function was observed in glaucoma patients who had a thinner lamina cribrosa [80]. As a thinner lamina cribrosa was independently linked to cognitive impairment in addition to the lower RNFL thickness, the authors suggest that the thinning of the lamina cribrosa may be a common mechanism driving both glaucoma and cognitive dysfunction.

On the other hand, it is crucial to recognize that the preservation of visual functionality plays a fundamental role in maintaining social integration and, consequently, the cognitive abilities of patients. Extensive evidence supports a direct link between sensory function and cognitive aging, emphasizing the role of sensory integrity in maintaining cognitive skills [81]. Furthermore, studies have shown that social disengagement represents a risk factor for cognitive impairment among elderly individuals [82], while active participation in cognitively stimulating activities is associated with a reduced risk of cognitive decline [83]. This holistic consideration of these patients cannot be overlooked in the study of glaucoma, making the research more complicated and highlighting the need for future investigations.

*Tau accumulation in glaucoma and glaucoma models.* These findings, together with the common features between neurodegenerative diseases and glaucoma, have led researchers to look for variations in the levels of certain specific characteristic proteins in the eyes of glaucoma patients. Among them, a few studies included the investigation of phosphorylated tau protein levels [51,52,84], suggesting that they may play a role in the degeneration of these cells. In 2005, Yoneda et al. measured tau protein concentrations in the vitreous fluid from patients with different ocular diseases, finding a significant increase in the tau level in patients with glaucoma compared with the control macular-hole patients [51]. In 2006, Oka et al. demonstrated a loss of tau proteins in the retina of mouse models with glaucoma [85]. The authors proposed that tau proteolysis may participate in the pathogenesis of neuronal cell death, correlating with an increase in calcium and calpain activation [85]. As the phosphorylation of tau is another factor causing neuronal cell death, the phosphorylated form of tau was investigated too, but it could not be directly detected due to its complete proteolysis. However, the authors suggest that it is indirectly evident from the upregulation of cyclin-dependent kinase 5 (cdk5) and p35/p25 [85]. Further, Tseng et al. hypothesized that the glaucomatous optic neuropathy originated from an accumulation of neurotoxic proteins, similarly to those found in Alzheimer’s disease [52]. The authors performed immunostaining for ß-amyloid, phosphorylated-tau, and α-synuclein in eyes with glaucoma and in a control group, finding similar levels of expression of ß-amyloid and α-synuclein in both groups, and higher levels of phosphorylated tau protein in glaucomatous eyes. Later, Gupta et al. demonstrated a decrease in normal tau protein in both the optic nerve and retina in glaucomatous patients with ocular uncontrolled hypertension compared with age-matched controls, and a significant increase in abnormal hyperphosphorylated tau protein, predominantly at the outer border of the inner nuclear layer [84]. They also analyzed eyes with incidental open-angle glaucoma (OAG), and did not find the abnormal tau protein. These results suggest that abnormal tau levels could be related to advanced glaucomatous damage [84]. Recently, Chiasseu et al. investigated the role of tau in the neurodegeneration of RCGs in an in vivo rat glaucoma model [4]. The study revealed a rapid increase in endogenous retinal tau with epitope-dependent changes in phosphorylation and tau oligomer formation as a result of increased intraocular pressure. Moreover, the authors also defended the crucial role of tau alterations in glaucoma-induced neurodegeneration, demonstrating that tau knockdown promotes robust RGC survival [4].

*Tau protein, axonal transport, and RGCs.* Furthermore, evidence suggests that tau protein may have a role in the regulation of axonal transport, which is essential for the preservation of RGCs and their axons [86,87]. Reduced axonal transport results in a state of cellular strain, contributing to neurodegeneration and limiting neurons’ ability to resist damage [54]. RGCs have been proven to be particularly sensitive to the impairment of axonal transport, as the functioning of these neuronal cells considerably depends on retrograde trophic support [88,89]. Thus, dysregulation of tau protein may impair axonal transport and consequent RGC degeneration in glaucoma. Bull et al. investigated the effects of tau hyperphosphorylation and aggregation on axonal transport in the optic nerve of mice transgenic for human mutant P301S tau, finding a significative reduction in both anterograde and retrograde axonal transport [54]. Moreover, the authors showed that the aggregation of mutant tau in RGCs was associated with an increased mild excitotoxic injury effect, resulting in greater nerve cell loss in the retina [54].

Stamer et al. studied the effect of microtubule-associated tau protein on the trafficking of vesicles and organelles in RGCs, finding that abnormally aggregated tau inhibits the kinesin-dependent transportation of mitochondria and peroxisomes toward the cell periphery, leading to a loss of energy production and the accumulation of reactive oxygen species [60,90].

*Tau protein, Aβ protein, and glaucoma.* Furthermore, tau protein has been shown to interact with amyloid beta (Aβ) protein, which is also implicated in the pathogenesis of glaucoma [85,91]. Pathological deposits of Aβ have been shown to be a cause of RGC death and thinning of the RNFL associated with glaucomatous degeneration [91]. Deposits of Aβ have been proven to be present in all retinal layers, including the ganglion cell, nerve fiber, and photoreceptor layers [60,92]. Recent evidence has reported that the Aβ protein plays a crucial role in controlling the post-translational modification of tau protein, promoting phosphorylation, and increasing the formation of tau species capable of aggregating in amorphous deposits and NFTs [91,93,94,95]. Chiasseu et al. investigated alterations in tau protein and gene expression, phosphorylation, and localization in mice models overexpressing mutant tau, finding a direct correlation between p-tau, Aβ deposits, and RGC death [96]. It is nonetheless intriguing to note that these findings do not align with the observations made by Tseng et al., who reported similar levels of expression for β-amyloid in both the non-glaucoma control group and the experimental group with glaucoma [52]. This discrepancy highlights the need for further investigation of the subject through additional studies.

*Genetic factors linking glaucoma and tauopathies.* In recent years, the potential for a genetic correlation between glaucoma and tauopathies has become a subject of investigation. Notably, in 2021, Gharahkhani et al. pinpointed three risk loci linked to AD and glaucoma (MAPT, CADM2, and APP) [97]. More recently, a systematic literature review uncovered 49 single nucleotide polymorphisms distributed across 11 risk loci associated with AD and glaucoma (AGBL2, CELF1, FAM180B, MTCH2, MYBPC3, NDUFS3, PSMC3, PTPMT1, RAPSN, SLC39A13, and SPI1) [98]. While this area of study remains ongoing, it provides further support for the notion of a connection between these pathologies.

## 5. Oxidative Damage Is a Common Feature in Both Glaucoma and Tauopathies

The data discussed above, together with similar clinical history features and concurrent disease onset, show that glaucoma exhibits pathological traits typical of tauopathies, including tau accumulation, impaired phosphorylation, dysregulation of axonal transport, and the interaction of Aβ deposits. However, glaucoma and other tauopathies also exhibit persistent DNA damage [99]. DNA damage response (DDR) kinases, such as ataxia telangiectasia mutated (ATM), which may phosphorylate various substrates, are activated in conjunction with the beginning of the lesions. Reactive oxygen species (ROS) and tau oligomers are likely responsible for the lesions [100].

In glaucoma, oxidative DNA damage is a pathogenic factor [101]. In fact, high levels of 8-hydroxy-2′-deoxyguanosine (8-OHdG) have been found in glaucoma patients’ aqueous humor and serum [101]. The DNA base 8-OHdG, which has undergone oxidative modification, is a sign of oxidative DNA damage [102]. The amount of 8-OHdG is significantly more prevalent in the trabecular meshwork in patients with open-angle glaucoma and positively correlates with rising IOP and deteriorating visual field [103]. Additionally, patients with glaucoma exhibit decreased base excision repair (BER) enzymes, such as poly (ADP-ribose) polymerase (PARP1), 8-oxoguanine DNA glycosylase (hOGG1), and X-ray repair cross-complementing group 1 (XRCC1), suggesting that a disturbance of DNA repair may be involved in the pathogenesis of POAG [104].

Additionally, POAG is linked to a rise in the amount of DNA breaks in the local trabecular meshwork and the regular circulating leukocyte [105]. Inflammation, apoptosis, senescence, and neural dysfunction can all result from the persistent activation of the DNA damage response, which can also lead to dysregulation of the cell cycle and re-entry into G1 [106,107].

In neurons of the lateral geniculate nucleus (LGN), primary visual cortex (V1), and secondary visual cortex (V2), laser-induced chronic glaucoma models in rhesus monkeys exhibited increased expressions of 8-hydroxyguanosine (8-OHG), indicating oxidative stress, and phosphorylated histone variant H2AX (γH2AX), indicating DNA double-strand breaks [108]. Interestingly, oral antioxidant supplementation and attenuation of DNA damage response following an optic nerve injury can reduce 8-OHdG, which is neuroprotective to retinal ganglion cells and encourages neurite regeneration [107].

In the hippocampus and frontal cortex, people with mild cognitive impairment or Alzheimer’s disease have more histone H2AX-positive neurons than age-matched controls [109]; people with neurodegenerative diseases also have more oxidative DNA lesions in their nuclear and mitochondrial DNA [110]. This increase could be explained by the ability of amyloid oligomers to reduce DNA-PK activity and BRCA1 protein levels [111].

These findings imply that DNA damage response, while supporting DNA stability and mutation prevention, may also contribute to the development of glaucoma and tauopathies [112]. Last, but not least, a vicious cycle between tau hyperphosphorylation and oxidative stress has been documented [113]. While oxidative stress can cause tau hyperphosphorylation, multiple investigations on various cellular or animal models of tauopathies have proven that the overexpression of mutant versions of human tau underpins certain types of tauopathies with dominant hereditary increases in oxidative damage. This gradual rise in ROS that correlates with illness development shows that ROS generation is only a byproduct of the pathophysiological process. Furthermore, hyperphosphorylation causes tau to aggregate [114] and spread in a way similar to that observed for prion proteins [115,116], further boosting ROS generation via NADPH oxidase [117,118].

This vicious cycle between tau hyperphosphorylation and oxidative stress [113], as well as tau aggregate formation, can induce glial inflammation (astrogliosis) and neuropathology in Alzheimer’s disease-related mouse models via innate immune sensors (receptors) such as Tool-like receptors (TLRs) [119]. In fact, tau-containing astrocytes have been discovered in numerous tauopathies, including glaucoma [120,121].

Of note, the resulting astrogliosis is a process associated with abnormalities of Aquaporin-4, also known as AQP-4, which is an encoded water channel protein, leading to disturbances of the glymphatic flow [122,123] which plays a crucial role in the removal of debris (including amyloid β (Aβ) and tau aggregates) across the neuronal interstitial space [124]. Interestingly, AQP4 abnormalities have been observed in tau-induced neurodegeneration [124], including glaucoma [125].

## 6. Oxidative DNA Damage and Tau

Tau is primarily considered a cytosolic protein, but it has been found to localize to the nucleus of both neuronal and non-neuronal cells and interact with nucleic acids [126]. Tau is hypophosphorylated in the nucleus of neurons [127], and in vitro research has demonstrated that tau binds to the minor grooves of DNA to shield it from ROS [128]. When hyperphosphorylated, it becomes less able to bind DNA [129].

Tau’s biochemical characteristics may be impacted by its interaction with DNA, promoting tau’s abnormal aggregation [130].

In physiological circumstances, tau binds chromatin in cultured murine primary neurons. Under stressful circumstances, this interaction can be altered due to its dynamic nature [131]. Tau most likely directly participates in DDR in cells in response to DNA lesions. In cells lacking tau, histone H2AX is more frequently phosphorylated on serine 139 (H2AX) [132], indicating either more DNA lesions or a slower rate of DNA repair.

When expressed in the nucleus, human tau can reduce DNA breaks caused by cellular stress compared to wild-type neurons [127]. In the hippocampus of animals, tau deletion also results in a slower rate of DNA DSB repair [132], pointing to a dual role for tau in the brain regarding DNA protection and direct or indirect DNA repair. To reiterate, chromosomal aberrations are present in the peripheral cells of patients with mutated tau [133]. Additional evidence for this comes from (i) the increased chromosomal aberrations in tau knockout mice and (ii) the higher risk of cancers, aside from tauopathies, in families with tau mutations [133].

**Figure 1:**
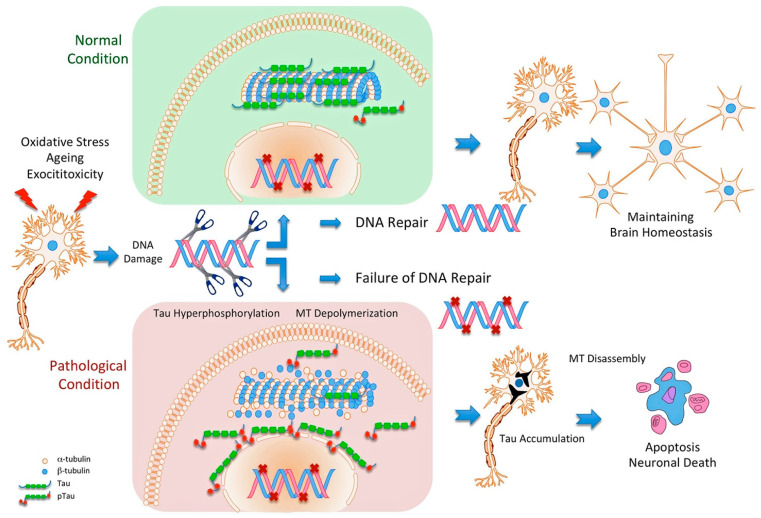
**General model of tau and DNA damage-induced neuronal death**. Following DNA damage, unphosphorylated tau (blue) physically binds to microtubules, causing microtubule (MT) polymerization and aggregation close to the perinuclear membrane and contributing to DNA damage repair (upper and green schema). Persistent DNA damage induces Tau phosphorylation and microtubules depolymerization. By inhibiting nuclear-cytoplasmic transport, accumulated p-tau (lower and pink schema) near the perinuclear membrane inhibits DNA damage repair, blocking the migration of damaged chromosomes or the recruitment of DNA damage repair proteins to the nucleus and leading to neuronal death.

Given what has been mentioned thus far, we suggest a model in which tau favors relocating damaged sites to perinuclear areas through enhanced microtubule polymerization, hence promoting proper DNA repair. High levels of oxidative stress (persistent DNA damage) cause the accumulation of p-tau, which impairs microtubule polymerization and the proper repair of DNA, resulting in cell death by starting a destructive feedback loop (Figure 1) [106,134].

## 7. Outlook for the Future

Intraocular pressure (IOP) is the only known major modifiable risk factor for glaucoma. Other non-IOP factors contributing to vision loss include neuroinflammation, oxidative stress, the dysregulation of calcium-dependent processes, defective autophagy, reactive gliosis, cribrosa translaminar pressure differences, and, possibly, the dissemination of misfolded proteins [135].

However, the reported accumulation of tau in the retina in glaucoma could be another factor contributing to vision loss, although the function of tau in glaucoma is still unclear.

There are currently no disease-modifying therapies for tauopathies available in Europe. However, potential therapeutic methods targeting various pathogenic pathways in tauopathies include the suppression of MAPT expression, regulation of alternative splicing, stabilization of microtubules, regulation of post-translational modifications, inhibition of aggregation, activation of tau clearance, and the use of passive or active immunization. These approaches aim to mitigate the harmful effects of tau and provide potential therapeutic strategies for tauopathies, and possibly glaucoma. Moreover, numerous laboratories have discussed the relationship between tau and the preservation of genome integrity [136]. Neuronal death and an accumulation of genomic lesions have been found in brain samples from various tauopathy patients. According to reports, aggregated and hyperphosphorylated tau may bind to and interact with cytoplasmic DNA repair proteins like BRCA1 to prevent DNA repair. However, evidence of a direct connection between tau, DNA damage, and neurodegeneration is still lacking.

Future research should examine how DNA lesions associated with glaucoma are created and repaired, including the effects of tau hyperphosphorylation, tau aggregation, and tau mutations. A better comprehension of the function of DNA lesions in glaucoma may also result in more effective and creative treatment plans.

Lastly, several other tauopathies may exhibit a common pattern of genomic integrity loss and persistent DNA damage caused by certain proteins that tend to aggregate abnormally, leading to neuronal death and impairing DNA repair mechanisms [137,138].

At this point, additional research is required to confirm and categorize tau pathologies and to better comprehend the mechanisms tying tau protein to the pathogenesis of tauopathies. However, the current results appear promising in substantiating this new theory.

## Data Availability

The data that support the findings of this review are available in the references cited within the article.
